# Precision Medicine for Acute Lymphoblastic Leukemia in Children: A Review

**DOI:** 10.3390/children11111329

**Published:** 2024-10-30

**Authors:** Anish Ray, Michael Levitt, Toluwalope Efunkoya, Heidi Trinkman

**Affiliations:** 1 Cook Children’s Medical Center, Fort Worth, TX 76104, USA; tolu.efunkoya@cookchildrens.org (T.E.); heidi.trinkman@cookchildrens.org (H.T.); 2 University of North Texas Health Science Center, Texas College of Osteopathic Medicine, Fort Worth, TX 76107, USA; michaellevitt@my.unthsc.edu

**Keywords:** pediatric acute lymphoblastic leukemia, precision medicine, targeted therapy, immunotherapy, pharmacogenomics

## Abstract

The clinical outcome for children diagnosed with acute lymphoblastic leukemia is a testimony to the success of modern medicine. Over the past few decades, survival has climbed from ∼10% to >90% for certain subgroups. Yet, the outcome for those with relapsed disease is often poor, and survivors struggle with a multitude of healthcare issues, some of which are lifelong. In recent years, the advent of the widespread sequencing of tumors has made available patients with previously unrecognized subtypes of leukemia, who have the potential to benefit from the addition of targeted therapies. Indeed, the promise of precision medicine, encompassing a person’s environment, genetics and lifestyle, is likely to have profound impact on further tailoring therapies that are likely to improve outcomes, diminish toxicity and ultimately pave the pathway for a healthier population.

## 1. Introduction

Over the last few decades, the overall survival for children diagnosed with acute lymphoblastic leukemia (ALL) has peaked to greater than 90% [1]. Such remarkable progress has been possible due to a multitude of factors, including the benefit of risk-adapted chemotherapy regimens, CNS-directed therapies, and multi-institutional collaborative randomized control trials, as well as significant improvements in supportive care measures such as the routine use of prophylactic antimicrobials [1]. Yet, successive trials conducted at Saint Jude Children’s Research Hospital (SJCRH) through studies 15 and 16 have demonstrated similar overall survival rates of 93.5% and 94.1%, respectively, suggesting that current risk stratification and chemotherapy-intensified regimens may have peaked in their efficacy [2,3]. On the other hand, there is significant unmet need concerning the preservation of health and medical complications among a growing population of childhood leukemia survivors. This creates an opportunity to explore the utility of therapies targeted at molecular abnormalities through the realm of precision medicine. In fact, embracing the universal genomic analysis of large groups of children and adults with ALL has re-shaped diagnosis, prognosis and therapy options for these patients (Table 1). Through the identification of unique clonal chromosomal alterations and their corresponding molecular expressions, there has been a significant shrinkage of patients belonging to an uncharacterized ALL subgroup from 25% to about 5% [4]. In fact, a timely confluence of technology, artificial intelligence and data-driven biology has created a perfect opportunity for a precision medicine-based approach; in addition, electronic health record systems have made it possible to combine and compare massive amounts of data that can be applied to improve patient outcomes.

## 2. Favorable and Unfavorable Genotypes in ALL: Past, Present and Future

Prior to advances in next-generation sequencing (NGS) technology, *ETV6::RUNX1*, *TCF3::PBX1*, hyperdiploidy (>50 chromosomes), and a trisomy of chromosomes 4, 10 and 17 were known markers of favorable outcomes, whereas the lysine methyltransferase 2A (KMT2A) (previously known as mixed-lineage leukemia or MLL) rearrangement, Philadelphia chromosome-positive (Ph+) (characterized by translocation 9;22), the intrachromosomal amplification of chromosome 21, and hypoploidy were considered unfavorable markers in patients with B-ALL. In recent years, however, the genomic landscape of both B- and T-lineage ALL has undergone rapid characterization, with the incorporation of gene expression-based subgroups in therapy decisions. Leukemia specimens can now be classified through whole-transcriptome sequencing, leading to the identification of aneuploidy or other chromosomal abnormalities, as well as the identification of genes, leading to the activation of tyrosine kinases. Such genomic alterations need to be considered alongside clinical features (such as age at presentation) and a minimal residual disease (MRD)-based response to therapy to optimize outcomes.

In addition to *ETV6::RUNX1* and hyperdiploidy in patients with MRD-negative status at the end of induction, two other B-ALL subgroups have emerged, both associated with favorable prognosis: those with an overexpression of *DUX4-* and *ETV6::RUNX1*-like gene expression profiles. However, since small numbers of patients were enrolled in these studies, further confirmation remains necessary [5,6].

## 3. Philadelphia Chromosome-Positive ALL

The treatment of Philadelphia chromosome-positive (Ph+) ALL with targeted tyro-sine kinase inhibitors (TKIs) marked a landmark moment in molecular-driven therapy for childhood ALL. Historically, Ph+ ALL was considered to be associated with dismal outcomes, with hematopoietic bone marrow transplantation being the best option prior to the advent of TKIs; despite this, 45% of patients achieved 7-year event-free survival [7]. However, the addition of a *BCR-ABL1* inhibitor, imatinib, to an intensified chemotherapy backbone, enabled a great majority of Ph+ ALL patients to avoid bone marrow transplant while maintaining a 5-year overall survival of 70% [8]. Subsequently, efforts were dedicated to overcoming mutations involving the ABL1 kinase domain, leading to a resistance to imatinib, eventually paving the way for the next-generation inhibitors dasatinib and nilotinib, with the former being able to cross the blood–brain barrier [9]. When compared with historical controls, dasatinib appeared to provide relative improved efficacy compared to imatinib. This was finally solidified when the Chinese Children’s Cancer group conducted a randomized controlled trial comparing the efficacy of imatinib at 300 mg/m squared per day against dasatinib at 80 mg/m squared per day in children with Ph+ ALL. The dasatinib group demonstrated significantly improved event-free and overall survival compared to the imatinib group, with neither group of patients receiving prophylactic cranial irradiation [10]. Subsequent trials are exploring the utility of third-generation ABL1 class tyrosine kinase inhibitors such as ponatinib (NCT04501614).

This discussion would be incomplete without mentioning one recent Italian study of 63 adult patients, with a median age of 54 years, with Ph+ ALL, who were treated without conventional chemotherapy. Instead, patients received induction therapy with dasatinib and glucocorticoids, inducing a 29% molecular response, which increased to 60% following two cycles of blinatumomab. At a median follow-up of 18 months, an overall survival of 95% and a disease-free survival of 88% were noted with very few toxic effects [11]. Whether such an approach can be replicated in children remains a matter of discussion of paramount importance.

## 4. Philadelphia Chromosome-like ALL

Activated tyrosine kinase gene expression profiles similar to Ph+ ALL were reported in 2009 by the Children’s Oncology group, University of New Mexico, and Saint Jude Children’s Research Hospital using the Affymetrix gene expression microarray. They, along with the Dutch Children’s Oncology group, almost simultaneously identified one subset of high-risk B-ALL patients presenting with high white-blood-cell count and advanced age as well as a tendency to respond inadequately to conventional treatment regimens [12,13]. This subgroup included variants involving *IKZF1*, *ETV6*, *ERG* and *PAX5* genes, leading to the activation of kinase signaling pathways that could be inhibited by specific TKIs. About 50% of these Ph-like ALL patients harbored cytokine receptor-like factor 2 (*CRLF2*) rearrangements activating the PI3K/mTOR and Janus kinase (JAK)-STAT pathways, most notably among Hispanic patients [14]. Those with JAK-STAT pathway activation responded to inhibitors such as ruxolitinib [14]. The remaining patients without *CRLF2* rearrangement comprised two main groups. The first group included patients with arrangements in *ABL1*, *ABL2* or platelet-derived growth factor receptor (*PDGFR*) alpha or beta. These patients are likely to respond to imatinib or dasatinib. The second group comprised patients with JAK-STAT signaling pathway mutations including JAK2 fusions [15]. There is a growing body of literature supporting the utilization of ABL- or JAK-directed tyrosine kinase inhibitors in combination with systemic chemotherapy to help improve the outcome of patients with Ph-like ALL [14]. Additionally, there is a smaller group of rare rearrangements involving the following genes: *NTRK3*, *PTK2B* and *TYK2,* among others [16]. While the investigation of targeted therapy against *TRK* fusions is currently underway (NCT03834961), further preclinical evidence is needed to elucidate the significance of the other rare rearrangements.

## 5. Infant ALL

Infant ALL has long been recognized for its unfavorable outcome. From a molecular standpoint, the most frequent rearrangement noted in this subgroup of patients is that of the *KMT2A* gene in about 70% of cases, with four other main partner genes (*AF4*, *AF9*, *AF10*, *ENL*) [17]. Despite the lackluster performance of lestaurtinib, an FLT3 inhibitor used with intensive chemotherapy in infant ALL [18], significant excitement has been noted around the development of menin inhibitors, presently in clinical development. Menin, a known tumor-suppressor gene in endocrine glands, has been associated with germline mutations causing multiple endocrine neoplasia type 1 (MEN1) syndrome as well as driving leukemogenesis in leukemia characterized by the rearrangement of the *KMT2A* gene [19]. This seemingly contradictory role highlights the many differing biological processes in which this gene is involved. The interaction of menin with KMT2A protein is considered responsible for the leukemogenesis in acute myeloid leukemia (AML) through the upregulation of the *HOX/MEIS1* genes [20]. Thus, menin inhibitors disrupt the menin-KMT2A complex, ultimately leading to the downregulation of *MEIS1* and *HOX*. In this regard, revumenib has demonstrated encouraging safety and efficacy when studied in AML patients with a rearrangement of KMT2A or mutation of nucleophosmin 1 (NPM1) [21]. Given that these molecular changes are also observed in patients with B-ALL as well as mixed-lineage acute leukemia, ongoing clinical trials are investigating the potential of revumenib in this subgroup of patients (NCT06575296).

## 6. Hypodiploid ALL

Hypodiploid ALL, comprising about 1–3% of childhood ALL, is associated with different genetic variations but universally poor outcomes [22]. Near-haploid ALL (<30 chromosomes) is characterized by RAS signaling pathway alterations and a high occurrence of *IKZF3* alterations, whereas low-hypodiploid cases (33–39 chromosomes) are associated with alterations of *RB1*, *TP53* and *IKZF2* and have a strong association with Li Fraumeni syndrome as well as secondary cancer [23]. The gene for *BCL-2*, or B-cell lymphoma/lymphoma 2 residing on chromosome 18, is associated with intrinsic cell death (apoptosis) and is hyper-expressed in ALL and AML, leading to the proliferation of cancer cells. The inhibitor of *BCL2*, venetoclax, has proven beneficial in the treatment of chronic lymphocytic leukemia and AML, as well as in in vitro studies of hypodiploid samples of ALL characterized by a high expression of BCL2 [24]. Gibson et al. from the MD Anderson Cancer Center identified 18 heavily pre-treated patients, comprised of B- and T-lineage acute lymphoblastic leukemias, between the ages of 6 and 22 years who were treated with venetoclax in addition to various combinations of concurrent chemotherapy [25]. Complete remission was achieved in 61% of the patients, with an overall survival of 9.1 months. These findings certainly provide encouragement for future combination therapies [25].

## 7. MEF2D-R ALL and HDAC Inhibitors

The rearrangement of myelocyte enhancer factor 2D (*MEFD2*) and other partner genes have been noted in about 3% of cases of B precursor acute lymphoblastic leukemia [22]. Among 107 MEF2D-r patients, although a majority were noted to have a good early response, at a median follow-up of six years, the event-free survival rate was recorded as 74%, thus confirming higher risk [26]. Early studies have indicated that *MEF2D* transcriptional activity may be sensitive to histone deacetylase inhibitors such as panobinostat [27].

## 8. T Lymphoblastic Leukemia

Genetic alterations in T-ALL have not added much prognostic value to MRD-based responses as they have for B lymphoblastic leukemias. However, the NOTCH pathway was noted to be most frequently dysregulated in T-ALL in a large group of 264 children studied by COG and SJCRH [28]. Unfortunately, inhibition of the NOTCH pathway by gamma secretase inhibitors, blocking an important structure in Notch1, was complicated by significant gastrointestinal toxicity as well as limited efficacy in some adult tumors, preventing further development [29]. The subsequent production of next-generation gamma secretase inhibitors remains in the developmental phase, while bortezomib, a proteasome inhibitor and sensitizer of traditional chemotherapeutics drugs, was studied in a phase III trial with a modified BFM chemotherapy backbone. Unfortunately, the addition of bortezomib did not demonstrate an improvement in outcomes among newly diagnosed T-ALL patients [30]. Immunotherapeutic interventions such as CAR-T therapy have revolutionized the outcome of B-ALL patients but have lagged in their impact on T-ALL, mostly due to the lack of a surface antigen being expressed exclusively on T leukemia cells and not on normal T cells. A strategy to overcome this is underway through the development of daratumumab, which targets CD38 preferentially expressed on T leukemia cells but not on normal T cells [31]. A phase II study of this drug in combination with chemotherapy is underway (NCT03384654).

## 9. Immunotherapy

Despite significant advancements in molecular therapies, approximately 15% of pediatric ALL cases harbor an unknown driver mutation or exhibit an identified target that is not currently actionable [32]. One method to target these ALL subtypes is immunotherapy, which is distinct from molecular approaches as it is agnostic to the underlying driver mutation. Rather, it relies on activating or facilitating pathways of the adaptive immune system to recognize and clear malignant cells. One such therapy that revolutionized the treatment of pediatric ALL is blinatumomab, a bispecific T-cell engager, consisting of two fragments: one that binds CD19 on B cells and another that binds CD3 and activates T cells. Blinatumomab has demonstrated an improved 2-year disease-free survival compared to standard chemotherapy in pediatric and young adult patients with Ph-negative B-ALL after first relapse [33]. Additionally, blinatumomab benefits standard-risk patients with bone marrow relapse, with an overall survival of 97.1% in those without extramedullary relapse [34]. These results have supported the recommendation of blinatumomab as a first-line treatment for relapsed/refractory B-ALL.

Inotuzumab ozogamicin, an antibody–drug conjugate targeting CD22, represents another promising immunotherapeutic option for patients with heavily pre-treated B-ALL. Upon binding to leukemic cells via inotuzumab, cytotoxic ozogamicin is internalized and released into the cell, inducing double-strand DNA breaks and eventual cell death. Two phase II trials utilizing inotuzumab ozogamicin have been conducted in pediatric B-ALL, both in significantly pre-treated diseases. The overall response ranged from 58.3% to 81.5% with one to two courses of treatment [35,36]. Though there were encouraging initial results in pediatric patients and promising responses in adult patients, further investigation of this antibody–drug conjugate remains a priority as it has not yet been trialed in larger studies, in combination with traditional chemotherapy, or in early disease in the pediatric population.

Chimeric antigen receptor (CAR) T-cell therapy has emerged as another revolutionizing advancement in the treatment of pediatric ALL. CAR-T cell therapy involves modifying a patient’s own T cells to express engineered receptors directed at a specific antigen. Tisagenlecleucel, directed at CD19 with a 4-1BB costimulatory signaling domain, is currently the only FDA-approved CAR-T cell therapy approved for use in pediatric patients [37]. In a phase II, single-cohort study, pediatric patients with CD19+ relapsed or refractory B-ALL treated with tisagenlecleucel had an overall survival of 90% at 6 months and 76% at 12 months [38]. Additional costimulatory signals such as CD28 have been trialed in pediatric patients alongside CD19-directed CAR-T cell therapy, with encouraging responses, but patients suffered from low rates of CAR-T cell persistence [39]. A positive response to CAR-T cell therapy relies upon the continued expression of the targeted antigen as well as a population of surviving CAR-T cells that provide ongoing surveillance. Therefore, efforts have been made to target other ALL-specific antigens such as CD22 to prevent relapse due to antigen escape. Complete remission was attained by 70% of patients in a trial utilizing CD22 CAR-T cells; however, due to concern of CD22 downregulation, this therapy is likely best utilized in the setting of HSCT to achieve an optimal response [40]. Treatment with CAR-T cells is associated with potentially life-threatening cytokine release syndrome (CRS) and, less commonly, neurotoxicity. CRS has been successfully managed with a single dose of tocilizumab, an anti-interleukin-6 receptor antibody [41].

While advances in the treatment of B-ALL with CAR-T cell therapy is promising, the treatment of T-ALL has proven to be more challenging due to a phenomenon known as fratricide. Fratricide occurs due to the expression of antigens that are shared between malignant T cells and CAR-T cells, resulting in the destruction of CAR-T cells by other CAR-T cells. However, advances in gene editing technology provide hope for fratricide-resistant CAR-T therapies for pediatric patients with T-ALL [42].

## 10. Pharmacogenomics

Pharmacogenomics has profoundly impacted the treatment of pediatric acute lymphoblastic leukemia, particularly in relation to thiopurine medications such as 6-mercaptopurine (6-MP). The discovery of genetic variants in thiopurine methyltransferase (TPMT) and nudix hydrolase 15 (NUDT15) has revolutionized how clinicians proactively manage thiopurine dosing to reduce toxicity and optimize efficacy [43]. Historically, TPMT was the first gene identified to influence the metabolism of thiopurines. Patients with reduced or absent TPMT activity are at increased risk for severe myelosuppression when treated with standard doses of thiopurines [44]. This discovery led to the incorporation of upfront TPMT pharmacogenomic testing as a standard practice in newly diagnosed pediatric patients with ALL, allowing for the individualization of therapy based on a patient’s genotype to prevent life-threatening toxicities [45].

More recently, the identification of NUDT15 as another critical gene affecting thiopurine metabolism, especially among Asian populations, has further refined treatment protocols. NUDT15 deficiency increases the risk of thiopurine-induced myelosuppression, independent of TPMT status, particularly with 6-MP [46,47]. The recognition of this variant has led to routine NUDT15 testing alongside TPMT to guide thiopurine dosing. This dual testing approach allows clinicians to pre-emptively adjust doses, minimizing the risk of severe toxicities while maintaining the therapeutic efficacy of treatment [48]. In practice, this pharmacogenomic-driven approach has reduced the occurrence of toxicities and improved outcomes in pediatric ALL, demonstrating the value of precision medicine in this population.

## 11. Conclusions

The landscape of pediatric ALL is undergoing a transformative shift, driven by advancements in identifying and targeting various ALL subtypes. Several pivotal events such as the availability of extensive and universal genomic sequencing as well as the rise of artificial intelligence in data analytics have ushered in a new and exciting era for B- and T-lineage ALL patients. Collaborative efforts that pool rare subsets of patients have also played a key role in advancing medical knowledge. Novel combinations of targeted therapeutics such as venetoclax and navitoclax hold remarkable promise alongside cellular immunotherapy, indicating a new chapter where therapy will be driven by molecular markers. In parallel, pharmacogenomic advances are enabling individualized drug dosing in an effort to maximize leukemic toxicity while minimizing off-target side effects. These approaches, in combination, will potentially enable us to avoid the toxicities of intensive systemic chemotherapy, thereby sparing long-term toxicities for survivors.

## Figures and Tables

**Table 1:** Targeted therapies for genetically defined subsets of pediatric acute lymphoblastic leukemia.

ALL Subtype	Targeted Therapy
Ph+ ALL	imatinib, dasatinib, nilotinib, ponatinib
Ph-like ALL	
with JAK-STAT pathway mutations	ruxolitinib
with ABL or PDGFR mutations	imatinib, dasatinib
with NTRK fusions	larotrectinib
KMT2A-rearranged ALL	lestaurtinib, revumenib
Hypodiploid ALL	venetoclax
MEF2D-rearranged ALL	panobinostat
T-ALL	daratumomab

## Data Availability

No new data were created or analyzed in this study.
